# Harmonizing yin and yang, remodeling the microenvironment: the adjuvant potential of Chinese herbal medicine in tumor immunotherapy

**DOI:** 10.3389/fonc.2026.1806602

**Published:** 2026-03-25

**Authors:** Xin Gao, Ying Han, Le Yang, Heng Fang, Hui Sun, Ye Sun, Guangli Yan, Ling Kong, Xijun Wang

**Affiliations:** 1 State Key Laboratory of Integration and Innovation of Classic Formula and Modern Chinese Medicine, National Chinmedomics Research Center, National Traditional Chinese Medicine (TCM) Key Laboratory of Serum Pharmacochemistry, Metabolomics Laboratory, Department of Pharmaceutical Analysis, Heilongjiang University of Chinese Medicine, Harbin, China; 2 State Key Laboratory of Dampness Syndrome of Chinese Medicine, The Second Affiliated Hospital Guangzhou University of Chinese Medicine, Guangzhou, China

**Keywords:** immune-related adverse events, immunogenic cell death, traditional Chinese medicine, tumor immunotherapy, tumor microenvironment

## Abstract

With immune checkpoint inhibitors (ICIs) and chimeric antigen receptor T-cell (CAR-T) therapy emerging as the fourth pillar of cancer treatment, modern oncology has entered a new era. However, clinical challenges—including primary/secondary resistance, limited response rates (particularly in “cold tumors”), and potentially severe immune-related adverse events (irAEs)—significantly constrain their applicability and patient benefit. Traditional Chinese medicine (TCM), grounded in its holistic principles of tonifying the body’s resistance while eliminating pathogenic factors and syndrome differentiation, demonstrates unique scientific value and translational potential through multi-component, multi-target synergistic actions. It remodels the tumor immune microenvironment (TME), enhances antitumor immune responses, and mitigates immunotherapy-related toxicities. This review systematically synthesizes current evidence elucidating core mechanisms by which TCM formulas, single compounds, and bioactive components enhance efficacy and reduce toxicity. Regarding efficacy enhancement, we focus on TCM’s role in reversing T-cell exhaustion, reprogramming tumor-associated macrophages (TAMs) and myeloid-derived suppressor cells (MDSCs), inducing immunogenic cell death (ICD), modulating tumor metabolic reprogramming, and optimizing gut microbiota composition to potentiate systemic antitumor immunity. For toxicity reduction, we comprehensively synthesize clinical evidence and pharmacological mechanisms underlying TCM’s mitigation of immune-related pneumonitis, colitis, cardiotoxicity, dermatotoxicity, and myelosuppression. This work establishes a robust theoretical foundation and scientific evidence for novel TCM-integrated strategies in cancer immunotherapy, while outlining future directions in the era of precision medicine.

## 1 Introduction

Cancer, as a persistent global public health threat, has undergone a profound therapeutic paradigm shift over the past decade. Immune checkpoint inhibitors (ICIs) and chimeric antigen receptor T-cell (CAR-T) therapy—representing immunotherapeutic strategies that reactivate the host’s endogenous immune system to recognize and eliminate tumor cells—have emerged as the fourth pillar of cancer treatment following surgery, radiotherapy, and chemotherapy, offering unprecedented survival benefits for patients with advanced-stage malignancies (1–3).

However, significant limitations persist in this revolutionary approach. Clinical evidence indicates that only a subset of patients derive therapeutic benefit, with a substantial proportion exhibiting primary resistance, while others develop acquired resistance following initial response (4–6). Immune-excluded “cold tumors,” characterized by an immunosuppressive tumor microenvironment (TME) with minimal T-cell infiltration, demonstrate minimal response to ICIs (7, 8). Critically, hyperactivation of the immune system can trigger immune-related adverse events (irAEs), affecting multiple organ systems—including cutaneous, gastrointestinal, pulmonary, endocrine, and cardiac tissues—with potentially life-threatening severity, representing a major clinical limitation for immunotherapy implementation (9–12).

Traditional Chinese medicine (TCM), an integral component of traditional Chinese medicine, has been widely used as supportive care in cancer treatment, particularly in East Asia. Core principles such as host resistance enhancement and pathogen elimination and holistic integration conceptually align with modern immunotherapy’s paradigm of systemic immune activation against localized tumors (13–15). TCM does not primarily exert direct tumoricidal effects but rather modulates systemic immune homeostasis—corresponding to the concept of yin-yang imbalance in traditional theory—through multicomponent, multitarget synergistic actions to remodel the immunological milieu conducive to antitumor immunity (16–18). Modern pharmacological studies initiated investigations into the immunomodulatory properties of herbal medicines as early as the late 1980s. For instance, Astragalus membranaceus extracts were demonstrated to enhance the cytotoxicity of lymphokine-activated killer (LAK) cells induced by subtherapeutic doses of interleukin-2 (IL-2), providing early experimental evidence for the immunomodulatory activity of traditional Chinese medicine (19, 20). In the 21st century, advances in systems biology, multidimensional omics technologies—including genomics, proteomics, metabolomics, and single-cell sequencing—coupled with artificial intelligence (AI), have enabled unprecedented depth and breadth in elucidating the complex mechanistic networks of TCM formulations and their bioactive components (21, 22). TCM modulates multiple critical the cancer immune cycle, including enhancing tumor immunogenicity, promoting dendritic cell (DC) maturation and antigen presentation, activating and recruiting effector T cells into the tumor parenchyma, and reversing immune-suppressive states within the TME (23–25). TCM achieves dual therapeutic objectives by enhancing immunotherapy efficacy while simultaneously mitigating irAEs through context-dependent immunomodulation (14, 26).

However, the current evidence remains fragmented, and a comprehensive synthesis of the mechanisms, clinical outcomes, and safety considerations of combining TCM with cancer immunotherapy is still lacking. Therefore, this review aims to systematically summarize available preclinical and clinical evidence on the adjunctive use of TCM in tumor immunotherapy. To improve transparency of evidence identification, we conducted a structured but non-exhaustive literature search in PubMed (supplemented by Web of Science and CNKI) from database inception to January 31, 2026. The PubMed search combined terms related to cancer immunotherapy and traditional Chinese medicine, including immune checkpoint inhibitors, CAR-T therapy, tumor microenvironment modulation, immunogenic cell death, immune-related adverse events, and representative herbal interventions. We specifically focus on its dual potential to mitigate immune-related adverse events and enhance antitumor efficacy, with the goal of providing a scientific rationale for the rational integration of TCM into contemporary immuno-oncology strategies.

## 2 Efficacy enhancement: multidimensional remodeling of the TME

### 2.1 Reversal of T-cell exhaustion and promotion of T-Cell infiltration

T cells, particularly cytotoxic T lymphocytes (CTLs), serve as the primary cytotoxic effectors in tumor cell elimination. However, in the TME, persistent antigen stimulation and inhibitory signaling are associated with T-cell dysfunction, inducing a state of exhaustion characterized by diminished proliferative capacity (meaningful reduction in clonal expansion), reduced cytokine secretion (substantial decrease in IFN-γ and TNF-α production), and elevated expression of co-inhibitory receptors (e.g., PD-1, CTLA-4, TIM-3, LAG-3). This exhaustion state is reported to be mechanistically sustained through TOX-mediated epigenetic reprogramming, which silences effector gene loci while promoting persistent expression of inhibitory molecules. Critically, exhausted T cells exhibit impaired metabolic fitness with significant reduced oxidative phosphorylation and glycolytic capacity, which has been reported to correlate with poor responses to ICIs in NSCLC and melanoma. Recent single-cell analyses reveal that TOX/NR4A transcriptional networks drive this dysfunctional state. These findings underscore the necessity of targeting exhaustion-specific pathways to rescue antitumor immunity., with elevated expression of co-inhibitory receptors (1). TCM may demonstrate multitarget intervention capabilities in reversing T-cell exhaustion.

Studies suggest that classical TCM formulas modulate T-cell subset homeostasis. For instance, in colorectal cancer mouse models, Dahuang Fuzi Baijiang Decoction potentiates anti-PD-1 therapy by restricting terminal exhaustion of CD8^+^ T cells while expanding progenitor-like T cells, a self-renewing subset with enhanced antitumor functionality (27). Classical TCM formulas—including Yangyin Fuzheng Jiedu Decoction, Yifei Sanjie Formula, and Xiaoji Yin—have been reported to attenuate the inhibitory effects of the PD-1/PD-L1 axis by directly downregulating PD-L1 expression on tumor and stromal cells, thereby facilitating T-cell reactivation (28, 29). Gujin Xiaoji Decoction potentiates anti-PD-1 immunotherapy in lung cancer by inhibiting the PI3K/AKT/NF-κB/PD-L1 axis (30).

**Figure 1:**
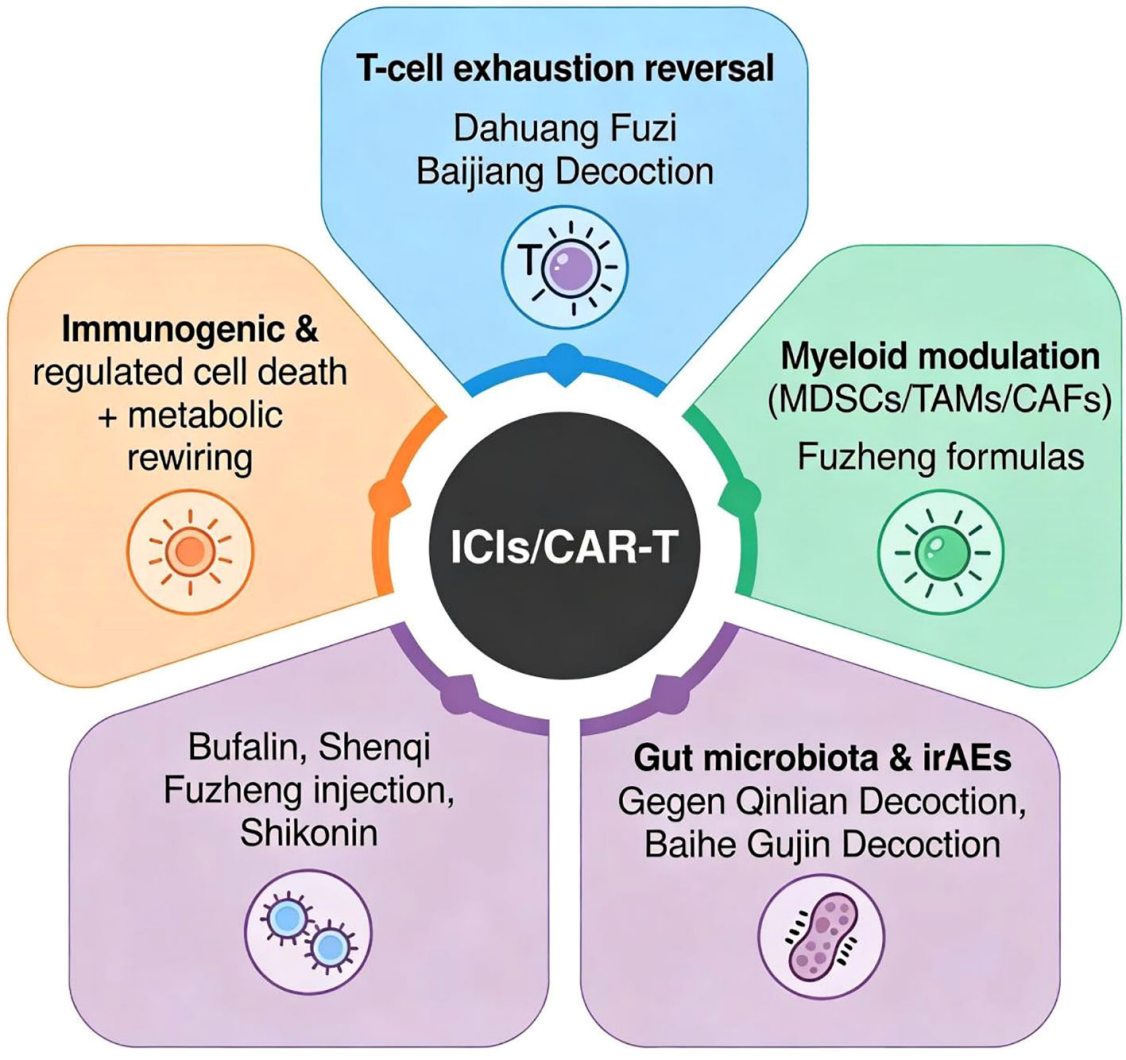
Overview of TCM-driven strategies enhancing ICIs/CAR−T therapy via T−cell exhaustion reversal, myeloid modulation, immunogenic/regulated cell death & metabolic rewiring, and gut microbiota & irAE mitigation (40).

Beyond formulations, bioactive monomers isolated from herbal medicines demonstrate potent T-cell regulatory functions *in vitro*/animal settings. For instance, icariin and baicalein potentiate cytotoxic T lymphocyte (CTL) infiltration and activation in tumor tissues by activating the STING (Stimulator of Interferon Genes) pathway to induce type I interferon production (31, 32). Tetrandrine potentiates anti-PD-1 therapy in non-small cell lung cancer (NSCLC) through activation of the STING/TBK1/IRF3 pathway (33). Luteolin enhances T-cell-mediated cytotoxicity by targeting peroxiredoxin 2 (PRDX2) (34). Ailanthone, a novel inhibitor targeting transcription factor c-Jun, downregulates PD-L1 expression in melanoma cells while reducing infiltration of immunosuppressive regulatory T cells (Tregs) in tumors, thereby potentiating anti-PD-L1 antibody therapy (8, 35). Additionally, preclinical studies have demonstrated that Huanglian Jiedu Decoction (36), Yadanzi Oil Emulsion (37), and Ginseng Polysaccharides (38, 39) enhance tumor sensitivity to ICIs through Toll-like receptor (TLR) pathway activation and other immunomodulatory mechanisms, see **Table 1** and **Figure 1**.

**Table 1:** TCM formulas and bioactive compounds that potentiate immune checkpoint blockade by reversing T-cell exhaustion and promoting T-cell infiltration.

TCM formula/compound	Modulatory mechanism & targets (keywords)	Summary of enhanced immune efficacy or effect	Key evidence/study
Dahuang Fuzi Baijiang Decoction	Limits terminally exhausted PD−1^hi Tim3^+^ T cells; expands PD−1^int TCF^+^ progenitor T cells; inhibits chemokine CCL2	Combined with PD−1 antibodies suppresses tumor growth, preserves progenitor T cells, and improves obesity-related microenvironment	In colorectal cancer models, the decoction combined with PD−1 blockade significantly prolonged survival and limited T-cell terminal exhaustion
Fuzheng formula/Yangyin Fuzheng Jiedu formula	Increases spleen and thymus indices; raises CD3+, CD8+ T cells; enhances intratumoral CD8+ T cells; inhibits PD−1, Tim−3, TIGIT; modulates PI3K/ERK/EGFR signaling	Significantly inhibits tumor growth (TGI ≈ 63%) and reduces multiple inflammatory cytokines in liver cancer models	Animal studies show the Fuzheng formula downregulates PD−1/Tim−3 expression and promotes apoptosis
Ginseng polysaccharides (GPs)	Modulates gut microbiota; increases short-chain fatty acids; raises CD8+/CD4+ ratio; increases sensitivity of non-responders to PD−1/PD−L1 antibodies	Combination with PD−1/PD−L1 antibodies significantly prolongs survival and inhibits metastasis; metabolites from altered microbiota enhance immune checkpoint blockade	Reviews note that GPs as adjuvants markedly increase ICIs response rates and improve immune microenvironment via gut microbiota remodeling
Baicalein and other flavonoids (e.g., ailanthone)	Downregulate PD-L1 or activate STING pathway; induce DNA damage and activate innate immunity	Described in the text as enhancing anti-PD−1 efficacy by lowering PD-L1 or activating cGAS-STING	Requires further clinical validation; mechanisms described without specific external references
Other compounds such as icariin, tetrandrine, salvianolic acid A	Modulate T-cell exhaustion or promote infiltration; mechanisms include inhibition of PRDX2, targeting ROS, etc.	Animal or cell models show reversal of T-cell fatigue and increased CD8+ T-cell proportion	Summarized from the original text; lacks systematic external research support

### 2.2 Myeloid-derived suppressor cells phenotype and function remodeling

The immunosuppressive state in the TME is maintained not only by T-cell dysfunction but predominantly by MDSCs and tumor-associated macrophages (TAMs) (23). These cells collectively establish an immunosuppressive network through inhibitory cytokine production (e.g., IL-10, TGF-β), depletion of T-cell-activating amino acids (e.g., arginine), and upregulation of co-inhibitory ligands (e.g., PD-L1).

TCM demonstrates significant efficacy in remodeling myeloid cells. While TAMs are polarized into proinflammatory M1 and protumoral M2 phenotypes, TME-resident TAMs predominantly exhibit M2 characteristics. Hugu Xiaoji Decoction (41), bufalin (42), Salvia miltiorrhiza extract (43), and Jianpi Huayu Decoction (44) effectively inhibit M2 polarization and repolarize TAMs toward the M1 phenotype. These agents block M2-mediated tumor angiogenesis, invasion, and metastasis by targeting key signaling pathways including Wnt1/β-catenin, TREM1/DAP12, and Cox2/PGE2. Similarly, huachansu and TCM formulations modulate TAM polarization to improve the immunological microenvironment (45).

Regarding MDSCs, their recruitment and expansion in the TME contribute significantly to immunotherapy resistance. Bushen Yiqi Formula (46), prim-O-glucosylcimifugin (47), and neobavaisoflavone (48) downregulate MDSC-recruiting chemokines (e.g., CCL2, CXCL1), reducing MDSC infiltration and breaking immunological tolerance. Shenqi Fuzheng Injection enhances anti-PD-L1 efficacy in melanoma brain metastasis models by modulating tumor fatty acid metabolism to suppress MDSC infiltration (49).

**Figure 2:**
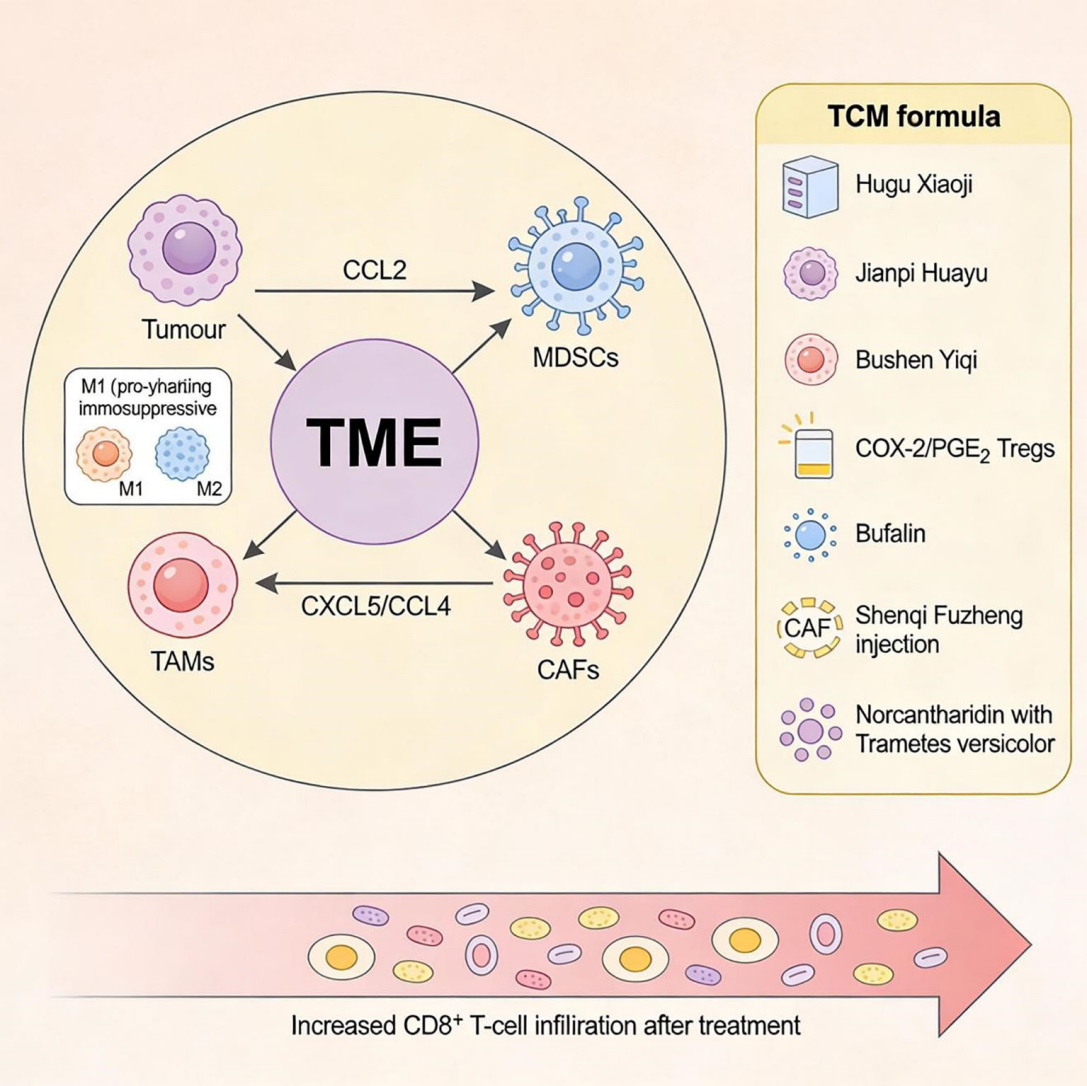
TCM−mediated remodeling of MDSCs, TAMs and CAFs in the tumor microenvironment. CAF, cancer-associated fibroblast; MDSCs, myeloid-derived suppressor cells; TAMs, tumor-associated macrophages; TME, tumor microenvironment.

Furthermore, classical TCM components including norcantharidin and Trametes versicolor extract inhibit cancer-associated fibroblasts (CAFs) through cytokine and chemokine modulation, thereby enhancing immunotherapy responses in hepatocellular carcinoma and triple-negative breast cancer (50, 51). See **Table 2** and **Figure 2**.

**Table 2:** TCM-mediated modulation of MDSCs, TAM polarization, and CAF-driven immunosuppression in the tumor microenvironment.

TCM formula/compound	Main targets & actions	Impact on immune microenvironment	Key evidence/study
Hugu Xiaoji Decoction, Jianpi Huayu Decoction, Bushen Yiqi formula	Inhibits MDSC recruitment, promotes MDSC differentiation; reduces M2 polarization of tumor-associated macrophages (TAMs)	These formulas are described as reducing MDSC numbers and enhancing antitumor immunity	Based on animal models and clinical observations, evidence is limited and mainly summarised
Bufalin (Huachansu)	Activates NF-κB signalling; drives tumour-infiltrating macrophages from immunosuppressive M2 to M1 phenotype; induces immunostimulatory cytokines	When combined with PD-1 inhibitor, can significantly inhibit hepatocellular carcinoma growth and enhance effector T cell responses	Studies show Bufalin drives tumour-infiltrating macrophages towards M1 polarisation, enhancing anti-tumour activity of PD-1 inhibitors
Danshen extract, Jianpi Huayu Decoction	Remodels TAM polarisation; induces IL-12 and reduces IL-10	Described as improving immunosuppressive microenvironment and enhancing antitumour immunity	Requires further external validation
Bushen Yiqi formula; prim-O-glucosylcimifugin; neobavaisoflavone	Inhibits chemokines related to MDSC mobilisation (such as CXCL5/CCL4), reducing MDSC content	Animal models show significant reduction in MDSC numbers and improvements in immunosuppressive status	Summarised from the original text; no clear external literature support
Shenqi Fuzheng Injection	Inhibits arachidonic acid metabolism in tumour cells; reduces COX-2/PGE2; decreases MDSC and Treg levels; enhances CD8+/CD4+ infiltration	Combination with PD-1/PD-L1 antibodies can enhance efficacy and improve the tumour microenvironment	Reviews indicate SFI modulates fatty acid metabolism and reduces immunosuppressive cells, thereby enhancing ICI effect
Norcantharidin; Trametes versicolor extract	Targets cancer-associated fibroblasts (CAFs); inhibits immunosuppressive factors induced by CAFs	Mentioned as intervening in immune evasion promoted by CAFs	Lack of publicly available clinical data

### 2.3 Induction of immunogenic cell death and activation of innate immunity

The mode of tumor cell death critically determines its capacity to activate antitumor immunity. Non-immunogenic forms of cell death (e.g., apoptosis) are immunologically silent, whereas ICD transforms dying tumor cells into endogenous “tumor vaccines” through the release of DAMPs—including CRT exposed on the cell surface, HMGB1, and ATP—thereby effectively activating dendritic cells (DCs) and initiating antigen-specific T-cell responses (52).

Numerous studies demonstrate that bioactive TCM components serve as potent ICD inducers. For instance, brucine induces ICD by disrupting lysosomal function (53), while shikonin (54) and licochalcone A (55) trigger ICD through enhanced endoplasmic reticulum stress. A combination of honokiol, magnolol, and baicalin remodels the colorectal cancer immunological microenvironment by triggering GSDME-dependent pyroptosis, exhibiting strong synergy with anti-PD-1 therapy (56). Similarly, bufalin nanoparticles induce pyroptosis via calcium overload.

Ferroptosis—an iron-dependent, lipid peroxidation-driven form of regulated cell death—has recently been identified as critically linked to antitumor immunit (57). Preclinical evidence indicates that artemisinin and its derivatives, along with specific flavonoids, can induce ferroptosis to release immunostimulatory signals, thereby synergizing with immunotherapy to enhance tumor killing.

By inducing these immunogenic cell death modalities, TCM not only directly eliminates tumor cells but also ignites immunological “ignition” at the tumor site, providing abundant “fuel” (tumor antigens) and potent “ignition signals” (DAMPs) for adaptive immune attack—critical for converting “cold tumors” into “hot tumors.”

### 2.4 Tumor metabolic reprogramming and gut microbiota modulation

Tumor metabolic reprogramming and gut microbiota modulation represent critical mechanisms in TCM-mediated immunotherapy enhancement. The TME exhibits a characteristic metabolic profile marked by hypoxia, acidosis, and nutrient depletion (e.g., glucose, glutamine), which severely impairs immune cell function—particularly T cells (58). TCM exerts systemic regulatory effects across multiple metabolic reprogramming axes. For instance, blood-activating and stasis-resolving formulations improve distorted tumor vasculature, alleviating TME hypoxia to enhance both drug delivery and immune cell functionality. Many TCM bioactive compounds directly target key tumor metabolic pathways, including glycolysis (Warburg effect), fatty acid synthesis, and amino acid metabolism. By inhibiting metabolic regulators such as HIF-1α, TCM reduces tumor hypoxia adaptation and synergizes with PD-1 inhibitors (59). Additionally, TCM modulates indoleamine 2,3-dioxygenase 1 (IDO1) activity to decrease immunosuppressive tryptophan metabolites (e.g., kynurenine), reversing T-cell suppression (60).

The ancient TCM concept of “lung-intestine axis” finds modern validation through gut microbiota (61). Gut microbiota not only regulates local immunity but also systemically influences antitumor immunity via metabolites (e.g., short-chain fatty acids, SCFAs) entering systemic circulation. Oral TCM formulations significantly reshape microbiota composition and function. Gegen Qinlian Decoction, Weidiao No. 3 Mixture (62), Shenling Baizhu Powder, and Zhenqi Fuzheng Granules (63) increase beneficial taxa (e.g., Akkermansia muciniphila, Bifidobacterium) while reducing pathogenic bacteria. This optimized microbiota profile elevates butyrate production, which enhances systemic type I interferon signaling and dendritic cell (DC) maturation via GPR109A activation, significantly improving PD-1/PD-L1 inhibitor efficacy in lung, liver, and colorectal cancers (64). This “gut-to-lung” or “gut-to-liver” immunomodulatory strategy provides robust mechanistic evidence for oral TCM formulations in the immunotherapy era see **Table 3**.

**Table 3:** Mechanistic summary of TCM-driven ICD, pyroptosis/ferroptosis, metabolic rewiring, and microbiome modulation in the tumor microenvironment.

Formula/compound	Mechanisms & keywords	Immune modulation & metabolic remodeling	Key evidence/study
Brucine, Shikonin, Licochalcone A	Induces multiple damage-associated molecular patterns (GRP78, HSP70/90, calreticulin, HMGB1) in tumor cells, promoting dendritic cell maturation	These DAMPs promote dendritic cell antigen presentation and activate effector T cells, resulting in immunogenic cell death (ICD)	Studies show that dendritic cell vaccines made from shikonin-treated tumor lysates enhance effector T-cell responses and significantly inhibit tumors
Magnolol/Honokiol/Baicalin combination	Induces GSDME-dependent pyroptosis; activates caspase-3 leading to GSDME cleavage	Pyroptosis releases cellular contents that enhance inflammatory responses, activate innate immunity, and synergize with PD-1 inhibitors; further research needed	The text describes this combination triggers inflammatory cell death; no external studies are cited
Bufalin nanomedicines, artemisinin derivatives, flavonoids	Induces ferroptosis; inhibits glutathione peroxidase 4 (GPX4); increases lipid peroxidation	Ferroptosis releases oxidized phospholipids, activates anti-tumor immunity; may improve immune checkpoint inhibitor response rate	Requires further experimental support
Metabolic reprogramming formulas (e.g., Guben Huanglian Jiedu Decoction, Zhimu Decoction)	Inhibit tumor glycolysis and fatty acid synthesis; decrease HIF−1α and IDO1 expression	Reduce immunosuppressive metabolites and enhance T-cell function	Described in the text; specific studies not cited
Gegen Qinlian Decoction, Weidiao No. 3 formula, Shenling Baizhu San, Zhenqi Fuzheng granules	Modulate gut microbiota; increase Bifidobacterium, Faecalibacterium, and butyrate-producing bacteria; promote production of butyrate and propionate	Remodelling microbiota can improve the tumor microenvironment and enhance the efficacy of PD−1/PD−L1 inhibitors; for example, Gegen Qinlian Decoction combined with PD−1 blockade can improve resistance	Clinical and animal studies indicate that Gegen Qinlian Decoction increases immune cell ratios and strengthens intestinal barrier function

## 3 Toxicity reduction: multitarget intervention of immune-related adverse events

### 3.1 Prevention and treatment of immune-related pneumonitis and colitis

Immune-related pneumonitis (CIP), characterized by excessive inflammatory infiltration in alveoli and interstitium, represents one of the most lethal irAEs (10). Clinical studies and case reports demonstrate that TCM adjunctive therapy significantly alleviates clinical symptoms (e.g., cough, dyspnea), improves pulmonary function, and may reduce corticosteroid requirements in CIP patients. For instance, Baihe Gujin Decoction has been validated in multiple studies to support lung cancer treatment, with its lung-moistening and yin-nourishing properties potentially mitigating pulmonary inflammation (65).

Immune-related colitis—a common severe irAE presenting with diarrhea, abdominal pain, and hematochezia—exhibits dual therapeutic mechanisms under TCM intervention: modulating intestinal immune responses to suppress excessive inflammation while repairing damaged mucosal barriers to promote tissue healing (9). A landmark multicenter randomized controlled trial (RCT) in NSCLC patients confirmed that Gegen Qinlian Tablets combined with ICIs significantly reduced overall irAE incidence compared to ICI monotherapy, with particularly pronounced efficacy in preventing diarrhea (Phase III RCT evidence) (26). Additionally, Sairei-to has been successfully employed to treat ICI-induced immune-related cystitis, demonstrating TCM’s potential in urinary system irAEs (66).

### 3.2 Mitigation of immune-related cardiac, cutaneous, and other systemic toxicities

Immune-related myocarditis, though less frequent, is highly lethal with elevated mortality (67). Xihuang Pills demonstrates cardioprotective effects in ICI-induced myocarditis models by modulating the HIF-1 signaling pathway, providing a novel strategy for prevention and treatment of this life-threatening complication (68).

Cutaneous toxicity represents the most common irAEs, presenting as rash, pruritus, and vitiligo, which may progress to Stevens-Johnson syndrome (SJS) or toxic epidermal necrolysis (TEN) (69). Clinical case reports confirm that topical Piyaning successfully mitigated SJS/TEN induced by ICIs, demonstrating TCM’s unique value in severe cutaneous toxicity. For common lichenoid dermatitis, Weiling Decoction also exhibits significant clinical efficacy (70).

TCM plays a critical role in managing other systemic toxicities. For chemotherapy- and immunotherapy-induced myelosuppression—particularly neutropenia and thrombocytopenia—tonifying formulas such as Buzhong Yiqi Decoction and Shenmai Injection significantly elevate white blood cell and platelet counts while reducing infection risk (71). For ICI-related fatigue, anorexia, and nausea, spleen-boosting formulas including Sijunzi Decoction and Liu Junzi Decoction improve digestive function and overall quality of life, enhancing patient tolerance to antitumor therapy (72).

TCM mitigates irAEs through multidimensional immunomodulation rather than non-specific immunosuppression like corticosteroids. By restoring immune homeostasis in a context-dependent manner, TCM effectively controls irAEs while preserving antitumor immunity—embodying the TCM principle of tonifying resistance without exacerbating pathogens, eliminating pathogens without impairing resistance. See **Table 4**.

**Table 4:** Traditional Chinese medicine–based supportive strategies for managing irAEs during immune checkpoint inhibitor therapy.

irAE type	TCM formula/compound	Health effects & mechanisms	Key evidence/study
Radiation- or immune-related pneumonitis	Baihe Gujin Decoction	Clears heat and nourishes yin; resolves phlegm and relieves cough. Clinical studies report reduced TGF−β1 levels, alleviation of interstitial lung fibrosis and inflammation, and improved quality of life. By modulating inflammatory and immune responses, it may help manage immune-related pneumonitis.	Reports indicate that Baihe Gujin Decoction combined with radiotherapy alleviated pneumonitis symptoms and lowered inflammatory markers such as CRP, PCT, and TNF−α.
Immune-related colitis/diarrhea	Gegen Qinlian (granules/tablets)/Xianglian pills; Saireito	Gegen Qinlian repairs the intestinal mucosal barrier, increases CD4^+^ T and NKT cells, reduces NF−κB and TNF−α, and remodels the gut microbiota. In murine colitis models, Saireito induces Th1 polarization and improves immune regulation.	Gegen Qinlian improved immune indices and intestinal barrier function in colorectal cancer patients; evidence for Saireito is mainly preclinical, with limited clinical irAE data.
Cardiotoxicity	Xihuang Pill	Described as promoting blood circulation, resolving stasis, and clearing heat/detoxifying; may alleviate immune-related cardiac adverse reactions through anti-inflammatory and antioxidant effects.	Primarily experience-based use; limited publicly available research evidence.
Dermatologic toxicity	Piyaning ointment; Weiling Decoction	Clears heat and detoxifies; dispels wind and relieves itching to reduce rash and pruritus; may modulate Th1/Th2 balance.	Traditional use and small observational studies; lack of large-sample evidence.
Myelosuppression	Buzhong Yiqi Decoction; Shenmai injection	Tonifies qi and nourishes blood to improve anemia and leukopenia; may restore bone marrow function by modulating the hematopoietic microenvironment.	Summarized as experience-based application; requires further clinical validation.
Fatigue/gastrointestinal symptoms	Sijunzi Decoction; Liujunzi Decoction	Strengthens the spleen and replenishes qi; harmonizes the stomach to relieve fatigue and anorexia.	Commonly used in clinical practice for chemotherapy/radiotherapy-associated GI discomfort; suggested as supportive care for irAEs.

## 4 Clinical evidence, future directions, and challenges

### 4.1 Accumulating clinical evidence

Clinical studies evaluating combined TCM and immunotherapy have increased significantly, particularly in China (73). In NSCLC, large-scale retrospective studies and meta-analyses consistently demonstrate that TCM adjuvant therapy—such as qi-nourishing and yin-nourishing or spleen-tonifying and kidney-supplementing approaches—combined with ICIs significantly prolongs PFS and OS while reducing irAEs incidence in advanced NSCLC patients (74). For instance, spleen-tonifying and qi-supplementing therapy significantly improves prognosis in ICI-treated NSCLC patients, while yin-nourishing and qi-supplementing formulations enhance PD-1/PD-L1 inhibitor efficacy.

In gastrointestinal malignancies, Huaier Granules demonstrates high-level clinical evidence for preventing recurrence in postoperative liver cancer and synergizes with targeted and immunotherapies (75). For colorectal cancer, Bazhen Decoction and Sijunzi Decoction enhance immunotherapy by improving immune function and quality of life (76). In gastric cancer, Liu Junzi Decoction alleviates chemoimmunotherapy-related gastrointestinal reactions and synergizes with primary regimens through TME modulation (72). Ongoing prospective RCTs evaluating Buzhong Yiqi Decoction or Shenling Baizhu Powder combined with ICIs in advanced solid tumors will provide further evidence for clinical implementation (77).

### 4.2 Future perspectives

To advance the precise and widespread application of TCM in tumor immunotherapy, future research must achieve breakthroughs in the following key areas:

First, biomarker discovery and integration—the cornerstone of modern translational precision syndrome differentiation. Future studies should establish deep correlations between TCM syndromes (e.g., qi deficiency, blood stasis, phlegm-dampness) and modern molecular biomarkers. For example, exploring intrinsic links between specific syndromes and tumor genomic mutation profiles, PD-L1 expression levels, tumor mutational burden (TMB), microsatellite instability (MSI), and the infiltration of specific immune cell subsets within the TME. This will facilitate the development of companion diagnostic biomarkers to guide TCM prescription, enabling personalized combined treatment strategies for each patient.

Second, development of novel drug delivery systems—addressing pharmacokinetic limitations such as low bioavailability and poor targeting of bioactive TCM compounds. Advanced nanotechnology offers an effective solution (78). For instance, liposomes, polymer nanoparticles, biomimetic nanovesicles (e.g., exosomes or cell membrane-coated nanoparticles) (79), or metal-organic frameworks (MOFs) can be engineered to encapsulate TCM monomers (e.g., curcumin, ginsenosides). These nanocarriers protect therapeutics from premature degradation and enrich drug accumulation in tumors via the enhanced permeability and retention (EPR) effect or active targeting strategies, significantly improving efficacy while reducing systemic toxicity. Notably, tumor cell membrane-coated nanoparticles delivering tanshinone IIA have demonstrated precise tumor targeting and high therapeutic efficacy (51).

Third, deep integration of multidimensional omics and artificial intelligence (AI)—essential for deciphering the complex “multi-component, multi-target” mechanisms of TCM formulations. High-throughput technologies including single-cell RNA sequencing (scRNA-seq), spatial transcriptomics, metabolomics, and proteomics provide unprecedented tools for systematic mechanistic analysis (21). These approaches enable precise mapping of dynamic changes in gene expression, functional states, and spatial distribution of immune and stromal cells within the TME at single-cell resolution following TCM intervention. Integration with AI and bioinformatics algorithms allows extraction of critical regulatory networks and targets from massive datasets, yielding deeper insights into TCM’s holistic immunomodulatory mechanisms (80).

### 4.3 Challenges and limitations

Despite promising prospects, the integration of TCM with immunotherapy faces significant challenges requiring urgent resolution. First, standardization and quality control remain critical barriers. Batch-to-batch variations in herbs like Astragalus membranaceus and Scutellaria baicalensis lead to inconsistent flavonoid and alkaloid profiles (e.g., significant variation in baicalin content), directly compromising biological activity and reproducibility in clinical trials (23). This heterogeneity impedes mechanistic validation of TCM-ICI synergy in NSCLC and HCC, where inconsistent TME remodeling (e.g., variable PD-L1 modulation) confounds efficacy assessment. To improve consistency, future studies should incorporate standardized sourcing and processing, multi-component chemical fingerprinting, and quantitative measurement of predefined marker compounds, ideally with batch-release criteria linked to bioactivity (81).

Second, mechanistic complexity necessitates deeper target validation. Current studies often lack rigorous confirmation of key pathways; only a small fraction of TCM research identifies specific molecular targets (e.g., STING/TBK1/IRF3) using CRISPR/Cas9 or gene knockdown validation, with most confined to correlative biomarker analysis without causal evidence. The transition from a “one drug, one target” model to a network pharmacology framework is essential to decipher the multi-target synergistic effects of TCM (82). Furthermore, the potential for herb-drug interactions (HDI) remains under-researched. Although ICIs (as monoclonal antibodies) generally have low potential for classic CYP-mediated pharmacokinetic drug–drug interactions, concomitant herbal products may still influence CYP enzymes, transporters, and gut microbiota-related metabolism, potentially affecting co-administered medications and overall treatment tolerability. Therefore, dedicated PK/HDI studies are needed, including CYP/transporter phenotyping, exposure-response analyses, and prospective safety monitoring in combination regimens. As TCM components may modulate cytochrome P450 enzymes or gut microbiota-mediated metabolism, their impact on the pharmacokinetics and toxicity profiles of ICIs requires systematic investigation to ensure patient safety.

Current evidence does not suggest that ICIs significantly alter the pharmacokinetics of concomitantly administered drugs. Therapeutic monoclonal antibodies, including PD−1/PD−L1 inhibitors, are eliminated via nonspecific catabolic pathways and do not directly interact with cytochrome P450 enzymes or drug transporters. Moreover, immunogenicity analyses show that anti−PD−1 antibodies such as nivolumab and pembrolizumab have minimal effect on cytokine levels that regulate CYP activity, indicating a low potential for pharmacokinetic drug–drug interactions. Prospective studies of cytokine−modulating biologics similarly demonstrate no clinically relevant changes in the exposure of probe substrates for CYP1A2, CYP2C9, CYP2C19, CYP2D6 and CYP3A. Consequently, regulatory guidelines do not currently recommend routine dose adjustments of small−molecule therapies when initiating ICIs, and real−world pharmacovigilance has yet to identify clinically meaningful CYP450−mediated interactions or alterations in the elimination half−lives of PD−1 antibodies. Ongoing vigilance and dedicated pharmacokinetic studies remain important as combination regimens evolve, but speculation without evidence should be avoided.

Third, high-quality clinical evidence remains scarce. While numerous studies exist, few meet the rigorous criteria for randomized controlled trials (RCTs), often suffering from inadequate blinding or single-center bias (5). To address this, international multicenter RCTs are now validating formulas like Baihe Gujin Decoction and Huaier Granules, focusing on survival endpoints (PFS/OS) and irAE reduction. A pivotal challenge lies in bridging traditional syndrome differentiation (Bian Zheng) with modern precision medicine. Future research should aim to identify “molecular syndromes”—linking TCM phenotypes (e.g., Qi deficiency, Blood stasis) with specific TME signatures or genomic profiles—to enable personalized integrative strategies. The adoption of Real-World Evidence (RWE) will also complement traditional RCTs by capturing the long-term benefits and safety of TCM in diverse patient populations.

Finally, regulatory and global integration represents a long-term goal. Aligning TCM’s holistic theory with international regulatory standards (e.g., FDA, EMA) requires interdisciplinary terminology and standardized manufacturing (ISO 13485). The integration of artificial intelligence (AI) and multi-omics will be transformative, allowing for the systematic mapping of TCM’s complex regulatory networks at single-cell and spatial resolutions (82). By fostering global collaboration among oncologists, immunologists, and TCM practitioners, we can establish a scientifically validated, patient-centered paradigm that addresses the current limitations of immunotherapy resistance and toxicity, ultimately improving outcomes for cancer patients worldwide (83).

### 4.4 Clinical trials of TCM combined with immunotherapy

To provide readers with a reference for the current clinical research landscape, we summarized the ongoing and recently completed trials evaluating TCM interventions combined with ICIs. These trials explore electroacupuncture or herbal formulas as adjuncts to ICIs in various malignancies such as early−stage or advanced NSCLC and recurrent/metastatic cervical cancer. **Table 5** outlines the trial phase, tumor type, primary endpoint and recruitment status to highlight the heterogeneity and current progress of this emerging field, see **Table 5**.

**Table 5:** Clinical trials of TCM combined with immunotherapy.

TCM intervention (trial)	Tumor type	Phase	Primary endpoint	Recruitment status
Electroacupuncture + ICIs (NCT07034326)	Early−stage NSCLC after surgery	2/3	2−year DFS	Recruiting
Bojungikki−tang + pembrolizumab (NCT06249854)	Advanced NSCLC (PD−L1 positive)	2	Progression−free survival	Active – Recruiting
Acupuncture + ICI (NCT06591078)	Recurrent/metastatic cervical cancer	N/A	Objective response rate	Active – Not Recruiting
Electroacupuncture + PD−1 inhibitor (NCT07086300)	Advanced NSCLC in the elderly	N/A	Progression−free survival	Recruiting

## 5 Conclusions

Cancer immunotherapy has fundamentally reshaped the therapeutic landscape for multiple malignancies; however, its clinical benefits are frequently constrained by immune-related adverse events and heterogeneous treatment responses. Current preclinical and clinical data indicate that TCM has the potential to mitigate immunotherapy-associated toxicities, including gastrointestinal, pulmonary, dermatologic, and systemic adverse events, thereby improving treatment tolerability and patients ‘quality of life. Beyond toxicity reduction, emerging studies also suggest that TCM may enhance the efficacy of cancer immunotherapy through multi-level immunomodulatory mechanisms, such as remodeling MDSCs phenotype and function, inducing ICD, activating innate immune responses, and modulating the gut microbiota–immune axis. These pleiotropic effects are particularly relevant in overcoming immune resistance and improving therapeutic responsiveness.

Despite these promising findings, the current body of evidence remains limited by heterogeneous study designs, small sample sizes, insufficient standardization of herbal formulations, and a lack of robust immune-related biomarkers. Therefore, high-quality, multicenter, and mechanism-oriented clinical trials are urgently needed to validate the safety, efficacy, and reproducibility of TCM combined with immunotherapy. Future research should integrate modern immunological assays, multi-omics technologies, and standardized TCM quality control systems to elucidate precise mechanisms of action and identify patient populations most likely to benefit.

In conclusion, TCM represents a promising adjunctive strategy in tumor immunotherapy, with the potential to both alleviate treatment-related toxicities and enhance therapeutic efficacy. With rigorous scientific validation and thoughtful integration into precision oncology frameworks, TCM may contribute meaningfully to the development of more effective, safer, and patient-centered tumor immunotherapy regimens.
